# A multi-component tailored intervention in family childcare homes improves diet quality and sedentary behavior of preschool children compared to an attention control: results from the Healthy Start-Comienzos Sanos cluster randomized trial

**DOI:** 10.1186/s12966-022-01272-6

**Published:** 2022-04-15

**Authors:** Kim M. Gans, Alison Tovar, Augustine Kang, Dianne Stanton Ward, Kristen Cooksey Stowers, Tayla von Ash, Laura Dionne, George Dennis Papandonatos, Noereem Mena, Qianxia Jiang, Patricia Markham Risica

**Affiliations:** 1 Department of Human Development and Family Sciences, University of Connecticut, 348 Mansfield Road, Storrs, CT 06269 USA; 2 Brown University School of Public Health, Box G-121-5, 121 S. Main St, Providence, 02912 USA; 3 Stanford University Medical School, Palo Alto, CA USA; 4 Department of Nutrition, Gillings School of Global Public Health, 135 Dauer Drive 245 Rosenau Hall, CB #7461, Chapel Hill, NC 27599 USA; 5 Department of Allied Health Sciences, University of Connecticut, Storrs, CT 06269 USA; 6 Rudd Center for Food Policy and Obesity, University of Connecticut, Hartford, CT 06103 USA; 7 Center for Health Promotion and Health Equity, Brown School of Public Health, Box G-121 8, Providence, RI 02912 USA; 8 Division of Nutritional Sciences, Cornell University, Ithaca, NY USA

**Keywords:** Randomized trial, Intervention, Childcare, Preschool children, Diet, Physical activity

## Abstract

**Background:**

Childcare settings are important environments for influencing child eating and physical activity (PA). Family childcare homes (FCCH) care for many children of low-income and diverse racial/ethnic backgrounds who are at greater risk for poor diet quality, low PA, and obesity, but few interventions have targeted this setting. The aim of this study was to assess the efficacy of a multicomponent intervention conducted in FCCH on the diet quality and PA of 2–5 year old children in their care.

**Trial design:**

Cluster randomized trial.

**Methods:**

The cluster-randomized trial, Healthy Start/Comienzos Sanos (2015–2019) evaluated an 8-month nutrition and PA intervention that included four components: (1) monthly telephone calls from a support coach using brief motivational interviewing, (2) tailored reports, newsletters and videos, (3) group support meetings, and (4) active play toys. After completing baseline measurement, FCCH were randomized into intervention or comparison groups in matched pairs. Both groups received the same intervention components but on different topics (intervention: nutrition/PA vs. comparison: reading readiness/literacy). Evaluation staff were blinded to group assignment. Child primary outcome measures collected at baseline and 8-months included: 1) Healthy Eating Index (HEI-2015) scores calculated from diet observation, and 2) accelerometer measurement of PA. Process measures were collected from field data and provider surveys. Generalized Estimating Equation Models assessed changes in HEI-2015 scores and PA over time by experimental condition.

**Results:**

Ethnically diverse FCCH providers (*n* = 119) and 2-to-5-year-old children in their care (*n* = 377) were included in the final analysis. Process evaluation showed high participation in all intervention components except for group meetings. Compared to children in comparison group FCCH, children in intervention FCCH increased total HEI-2015 scores by 7.2 points (*p* < .001) including improvement in component scores for vegetables (0.84 points, *p* = .025) and added sugar (0.94 points, *p* = .025). For PA, compared to children in the comparison group, children in intervention FCCH decreased sedentary time by 5.7% (*p* = .021).

**Conclusions:**

The multicomponent Healthy Start intervention was effective in improving diet quality and sedentary behavior of children in FCCH, which demonstrates the promise of obesity prevention interventions in this setting. Future research could include enhancing the Healthy Start intervention to strengthen the PA component, considering virtual peer support, and determining how to best translate and disseminate the intervention into FCCH nationally.

**Trial registration:**

National Institutes of Health, NCT02452645. Registered 5 May 2015.

**Supplementary Information:**

The online version contains supplementary material available at 10.1186/s12966-022-01272-6.

## Background

Globally, the prevalence of overweight and obesity in young children is high, with 38 million children under the age of 5 affected by overweight or obesity in 2019 [1]. In the United States, 13.9% of 2–5 year-old children are classified with obesity [2] with young children from low-income and racial/ethnic minority families even more likely to experience obesity [2, 3]. For example, low-income Latinx children are at much higher risk (25.8%) compared to their non-Latinx white peers (14%) [1, 2]. Obesity in childhood is associated with a wide range of adverse health outcomes [1, 4–9] over the life course, increasing the lifetime risk of many chronic diseases [9–12]. Thus, combating childhood obesity is a public health priority and primary prevention is needed.

The main lifestyle determinants of obesity are poor diet and physical inactivity (including excessive sedentary behavior) [8, 13]. In addition to influencing weight, a high-quality diet and regular physical activity (PA) improve young children’s development as well as physical and mental health [14–17]. However, the majority of young US children do not meet guidelines for healthy eating, PA, and sedentary behavior [18, 19]. These behaviors are adopted early in life and childhood obesity often starts before age five [20, 21]; thus, it is important to intervene early while children are still developing dietary and activity habits.

Childcare settings provide a valuable opportunity to promote healthy eating and PA because a significant proportion of young children spend time in childcare for prolonged periods throughout the day [22–24]. In the U.S., approximately 80% of preschool-aged children with working parents are in some form of childcare [23, 25–27], where they spend on average 22.5 h per week [28, 29], and may consume 50–70% of their daily food intake [24, 30–32]. Furthermore, childcare settings have a substantial influence on PA levels during the day [33, 34].

Thus, interventions to improve children’s and PA behaviors in childcare settings are greatly needed [35–38], especially among providers who serve low-income, ethnically diverse families who are at higher risk for developing obesity. Interventions have primarily focused on center-based care [39], with few interventions in family childcare homes (FCCH) also called family day care [40], even though 26% of US children in childcare—nearly 2 million young children—are cared for in such settings [41–43]. FCCH are an appealing option for low-income families as they offer a more intimate setting with fewer children, often provide flexible hours, and may be more affordable [44]. Thus, many FCCH providers care for low-income, ethnic minority children and are often themselves low-income and ethnically diverse [43, 45–48]. For example, in Rhode Island (RI), a state where approximately 16% of the population identifies as Hispanic or Latinx [49], at least 40% of FCCH providers (FCCP) are Spanish speaking [47].

The FCCH environment can be quite different from centers. Centers typically divide children into different groups based on age whereas FCCH take care of children of varied ages, so they have to arrange meals and activities that accommodate children at different developmental stages at the same time [39, 40, 50]. FCCH also tend to have less structured schedules and operate with different logistical and space constraints than centers. Finally, FCCH have different regulatory standards for nutrition and PA than childcare centers [47, 48, 51–53] and FCCP may feel isolated with regards to training, resources and technical assistance [48].

While less obesity-related research has been done in FCCH, a number of US studies have reported that children’s diet quality in FCCH needs improvement [40, 47, 54, 55]. Furthermore, reviews across studies indicate that preschoolers’ PA levels in FCCH are lower [40] and screen-time is higher [40, 56] than national recommendations. Additionally, time spent in FCCH during infancy has been associated with increased weight at one and 3 years of age [57], thus making FCCH an important target for early childhood obesity prevention efforts. A 2020 systematic review conducted by Yoong et al. identified and assessed the effectiveness of interventions to improve the diet intake, PA and weight status of children aged 0–6 years attending FCCH [50]. They found only two intervention studies with quasi-experimental designs [58–62] that examined changes in FCCH food and/or PA environments, but no randomized trials nor studies that examined child-level outcomes. These findings clearly indicate a need for controlled trials to identify effective obesity prevention interventions in FCCH. Since that publication, one recent high quality cluster-randomized intervention trial in North Carolina FCCH resulted in improvements in children’s diet quality [63]. However, additional robust studies are still needed in FCCH with diverse populations [50, 63].

The purpose of this paper is to present child-level outcomes from the Healthy Start/Comienzos Sanos study [64], a cluster-randomized trial evaluating the efficacy of a multicomponent intervention to improve the food and activity environments of FCCH, as well as the diet and PA of the 2- to 5-year-old children in their care [55, 64–68]. Study hypotheses relevant to the current analysis are as follows: 1). Intervention group children will improve their diet quality (Healthy Eating Index) at the FCCH more than comparison group children; 2). Intervention group children will improve their accelerometer-measured PA at the FCCH more than comparison group children.

## Methods

The methods of Healthy Start/Comienzos Sanos have been described in detail elsewhere [64], but those relevant to the current analyses are described briefly below. The Institutional Review Boards of Brown University, University of Connecticut and University of Rhode Island approved all study procedures and materials. Providers and parents provided written informed consent prior to measurement.

### Formative research

The Healthy Start study was informed by a state-wide survey with 105 FCCP in RI (39% Latinx) [69] as well as 7 focus groups with 51 participants (100% female, 88% Latinx) [70]. The findings from this formative research informed the adaptation of evaluation measures and the nutrition and PA intervention to address knowledge, skills, attitudes, beliefs, and barriers described by the FCCP. In addition, cognitive assessment testing was conducted with 6 FCCP (67% Latinx) to assess comprehension, terminology, and culturally appropriateness of the evaluation surveys. The information collected was used to finalize revisions to the baseline evaluation measures prior to their use with study participants.

### Trial design

Healthy Start was a cluster-randomized trial.

### Study settings and participants

Recruitment, enrollment and baseline assessment was conducted on a rolling basis from 2015 to 2018. A variety of recruitment strategies were used including: (1) Information sessions at community organizations that provide training and support for FCCP. These organizations also offered recruitment flyers and brochures to FCCP; (2) Meetings with the coordinators of FCCP systems who then emailed study information to FCCP in their systems; (3) Presentations at local FCCP conferences; (4) Direct mailings followed by staff phone calls to licensed FCCP whose contact information was publicly available through state databases in RI, and Massachusetts (MA); and (5) Word of mouth referrals from FCCP already participating in the study.

Interested FCCP were then contacted by research staff via telephone to assess eligibility. To be eligible, FCCP had to meet the following criteria: (1) Have a FCCH within 60 miles of Providence, RI in operation for at least 6 months; (2) Be able to read and speak Spanish or English; and (3) Care for at least one unrelated 2–5 year-old child for 10 h or more per week who ate at least one meal and snack per day at the FCCH.

Eligible providers completed a 30-min baseline telephone survey, followed by a 30-min in-person survey at the FCCH. FCCP provided written informed consent for their study participation. Consent forms for children to participate in the evaluation were then distributed to parents at the FCCH. To participate, children had to be aged 2–5 years, attend the FCCH for at least 10 h per work, and the study had to receive written consent from the child’s parent to have their diet observed by project staff, wear an accelerometer activity monitor and/or undergo anthropometric measurements. When at least one parent of an eligible 2- to 5-year-old child consented, a two-day observation was scheduled. Parents could consent for their child to be observed during mealtimes, to wear an accelerometer and/or have their anthropometric measurements taken. Participating FCCP received $25 for completing the baseline survey and $50 for the two-day observation. Children received a reusable water bottle as a thank you gift and parents received a $20 gift card. At the end of the 8-month intervention, surveys and two-day observations were repeated.

### Relevant measures

On the telephone survey, FCCP reported their gender, ethnicity, and race. The following variables were collected on the in-person survey: age, household income, marital status, education, years in the United States, country of origin, years as a child care professional, number of children currently in their care (and how many were their own children or grandchildren), and whether the FCCH was enrolled in the Child and Adult Care Food Program (CACFP), a federal program that provides reimbursements for nutritious meals and snacks to income-eligible children who are enrolled for care at participating childcare facilities.

The two-day observation included the Environment and Policy Assessment and Observation (EPAO) [71] and the Dietary Observation in Child Care (DOCC) [72]. The EPAO was used to evaluate the FCCH food and PA environment including FCCP behaviors and FCCH physical spaces and equipment (not included in this paper), while the DOCC was used to assess child-level dietary intake. DOCC is a reliable, valid visual observation technique for measuring children’s dietary intake [72]. The two observation days, which were usually, but not always consecutive, were scheduled at the convenience of the FCCP, as well as anticipated availability of the consented children. Staff members arrived before the first meal or snack was served at the FCCH. Observers positioned themselves to be able to observe up to 3 children in a convenient location to avoid distracting children or interfering with the daily routine. If more than 3 children were consented to participate, more than one research staff member conducted the observation so that no one observed more than 3 specific children. Observers left the FCCH during the children’s naptime and returned to continue with observation until the consented children left the FCCH to go home.

Data collectors underwent extensive training and certification prior to field work as well as continued quality control checks during the study [64]. The certification process for DOCC included a lab component, during which field staff had to accurately estimate at least 80% of 20 measured portions of food that a 2–5-year-old child would typically eat. After passing the lab certification process, field staff also had to achieve 80% inter-rater reliability with a “gold standard” observer in the field at a FCCH. DOCC observers had to pass the certification process annually, as well as participate in structured monthly practice, quarterly validity checks, and semi-annual inter-rater reliability checks. Quarterly, all observers repeated the certification process comparing estimates to measured food portions.

In the FCCH, data collectors observed all meals/snacks during the observation period over 2 days and carefully recorded food served, asking the provider for brand or recipe specifics, and watched and estimated the amount of food and beverages served, wasted (e.g., dropped or traded, etc.), and remaining. The amount of food consumed was estimated as the amount served minus the amount wasted or remaining for each child [72]. This recorded meal data was later processed by a trained data analyst as “per child” food intake data.

*Accelerometer Measurement of Children’s Physical Activity:* Children’s PA was assessed with Triaxial GT3X™ Actigraph accelerometers for 2 days during childcare. At the start of each day, one dedicated data collector placed the accelerometer on a belt around the waist of assenting children who had written parental consent. For most children, accelerometers were worn all day, removed by data collectors before the child left to go home. The accelerometer was worn during nap time unless the child was uncomfortable, in which case it was removed and put back on when the child woke up. After children had worn the accelerometer for both observation days, the accelerometer data were uploaded for processing.

The same research staff who attached accelerometers also measured consented children’s height, weight, and waist circumference using standard techniques [73]. Height was measured using a SECA portable stadiometer to the nearest 8th of an inch. Weight was measured using a Tanita digital scale to one decimal place. Waist circumference was measured to the nearest 8th of an inch by holding a standard tape measure around the child’s waist parallel to the floor at the top of their right ilium. The series of three measurements was repeated 3 times and averaged for each child. Weight status was calculated as age and sex specific Center for Disease Control (CDC) z-scores [74]. Overweight was defined as per the CDC as BMI > 85th percentile and obesity as BMI > 95th percentile [74, 75].

### Randomization

Once FCCP completed all baseline measures, they were randomized into either the Intervention or Comparison group in matched pairs based on primary language spoken and number of age eligible children in their FCCH using a Microsoft Excel randomization function. They were then notified of their assignment by a phone call from the project coordinator. Evaluation staff members were not informed of the intervention group assignment.

### Intervention

**Fig. 1:**
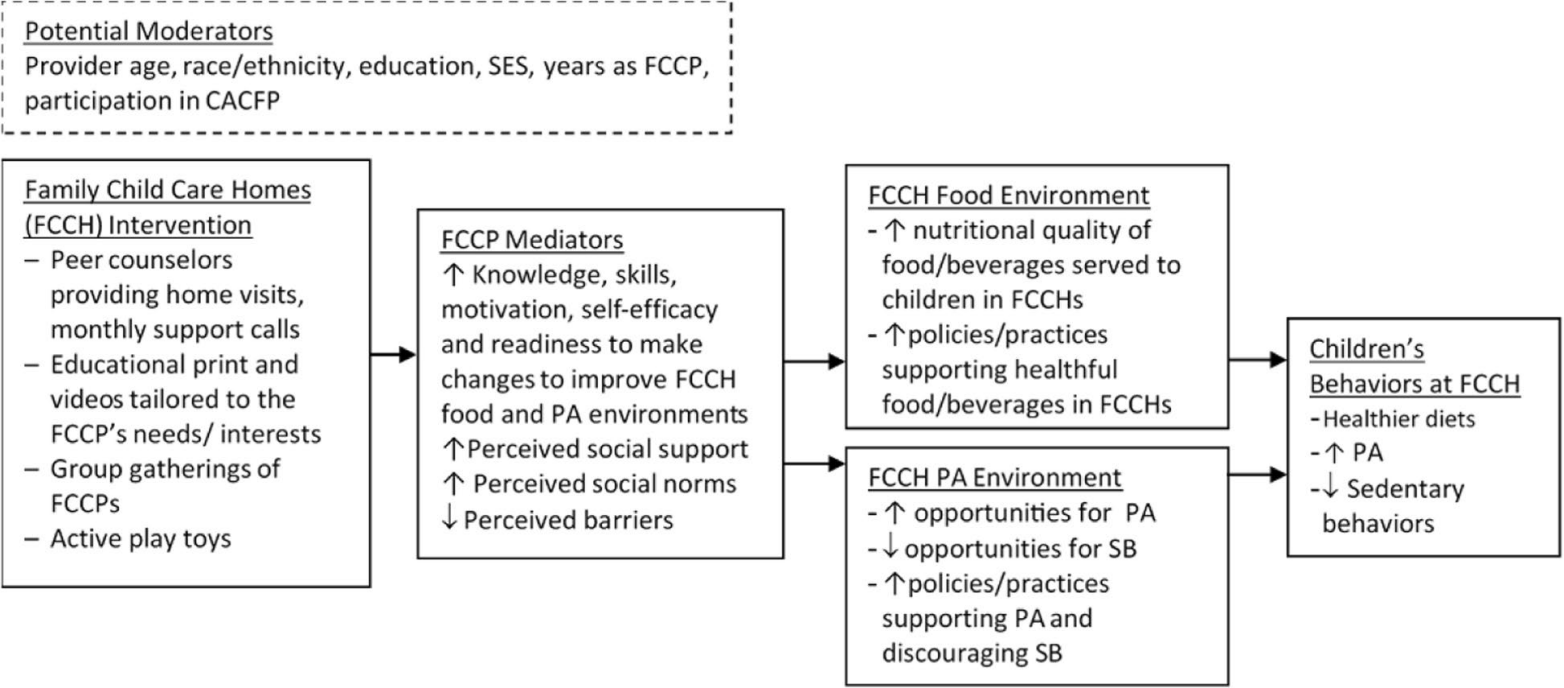
Logic Model of the Intervention

Theoretical framework: The Healthy Start intervention was informed by the social ecological framework, which recognizes that behavior is affected by multiple levels of influence and that interventions are most effective when they target changes at more than one level [76–81]. The intervention aimed to improve FCCP nutrition- and PA-related behaviors, which were then expected to result in changes to the FCCH nutrition and activity environment that would in turn lead to improvements in children’s diet, PA and sedentary behaviors. The intervention was also informed by Social Cognitive Theory (SCT), which defines behavior as a dynamic and reciprocal interaction of personal factors, behavior and the environment [80–83]. The intervention targeted key components of the SCT to change FCCP’s behavioral capability, self-efficacy, outcome expectations, perceived social support, norms, and barriers that would in turn lead them to improve their nutrition and activity-related practices and their FCCH’s environment to better support children’s healthy eating and PA. Moreover, as posited by Self Determination Theory [84, 85], motivational interviewing (MI) used by the support coaches was expected to increase FCCP’s motivation and readiness to change. (See Logic Model in Fig. 1).

The intervention is described in detail elsewhere [64]. Briefly, the eight-month intervention included four components: 1) Monthly support from a support coach; 2) Tailored materials including a tailored report, newsletters and videos; 3) In-person group meetings; and 4) A set of active toys. FCCP in the intervention group were assigned a lay support coach who was trained in brief MI and either the nutrition/PA content or comparison group literacy/reading readiness content. The intervention began with an in-person visit at the FCCH led by the support coach in which the coach reviewed with the FCCP an individually tailored written feedback report that indicated whether the FCCP met or did not meet best practices for 22 nutrition, PA and screen-time topics based on baseline data. These best practices were based mainly on the Nutrition and Physical Activity Self-Assessment for Child Care (NAP SACC) recommendations [86–88]. See Appendix 1. The coach then conducted brief MI [89] with the FCCP. At the end of the MI session, the FCCP selected one topic to work on. See Appendix 1 for example pages of the tailored feedback form.

At the in-person coach visit, the FCCP in the Intervention group also received a set of active toys (e.g. hula hoops, tunnel, bean bags, soft balls) with accompanying activity cards and video clips with ideas for using the toys. One to 2 weeks later, the FCCP received a tailored mailing in English or Spanish including a cover letter, newsletter and DVD (or emailed video link) tailored to the topic chosen above. The cover letter was micro-tailored with the names of the FCCP and their support coach, the FCCP’s topic choice and their baseline practices related to this topic, the FCCP’s chosen goal, a brief description of any expressed barriers, comments gathered from the last support coach session, and the scheduled day/time for the next support coach call. The newsletter included 4–5 pages about the FCCP’s chosen topic. If the FCCP mentioned a barrier (e.g. time, cost, taste, resources needed) during the coaching session, they also received 1–2 tailored pages with suggestions on how they might overcome the barrier. The 3–6 min video demonstrated best practices on the topic, testimonials, cooking and/or activity demonstrations, and scenarios displaying problem-solving.

Approximately 1–2 weeks after that mailing, the support coach called the FCCP and again used MI to discuss progress, and to help the FCCP select the next topic to work on. For the next 7 months, FCCP were sent a monthly tailored packet (letter, newsletter and video) based on the monthly topic they chose during their monthly support coach MI-based phone calls. In addition, group support meetings were held approximately every 6 weeks separately for the Intervention and Comparison groups in a central public location like a library or church. All participating FCCP were invited to attend these meetings, led by the support coaches, to support one another, discuss challenges and successes, learn a new activity, and share a meal.

### Comparison group intervention

FCCP randomized into the comparison group received the same intervention components, at the same dose and intensity as those in the intervention group, except the content was related to reading readiness and early literacy skills. The comparison group components included a tailored feedback form, an in-person home visit and seven monthly calls from the support coach using MI 8 tailored newsletters and videos mailed monthly, and the group meetings (approximately every 6 weeks), with content related to reading readiness and early literacy skills rather than nutrition and PA. The intervention content was adapted from the Reading Rockets and Coloring Colorado curriculum materials [90, 91]. FCCP in the comparison group received a set of 10 books in English and/or Spanish instead of the active toys.

### Process evaluation

Process evaluation measures assessed fidelity and dose of intervention delivery as well as FCCP satisfaction with the intervention. All in-person meetings and phone calls with support coaches and FCCP were digitally recorded, with at least 10% of the recordings reviewed by the project coordinator to assess support coach fidelity to intervention protocols. Support coaches also recorded attempts to connect with FCCP and completed forms to document what was discussed on each call. For dose, we measured coach-reported completions of in-person meetings and phone calls with FCCP as well as FCCP-reported number of newsletters and videos read and watched, group meetings attended, and toys/books used. FCCP satisfaction with each intervention component was assessed on the follow-up survey.

### Effect evaluation at the child level

*Primary outcomes:* Primary outcomes were changes in children’s diet quality and changes in children’s PA. Changes were calculated based on cross-sectional data collected at each time point, not paired differences among the same group of children. Diet quality was assessed using the 2015-Healthy Eating Index (HEI) score, calculated based on data collected during the two-day observation using the DOCC. DOCC data collected in the field were reviewed for completeness and entered into the Nutritional Data System for Research (NDSR), which translates food quantities into food and nutrient variables for analysis.

For each child, data were summed across both observation days, then the HEI-2015 algorithm was applied to calculate HEI total and component scores [92]. HEI scores assess compliance with national dietary guidelines. HEI component scores are calculated as intake per 1000 cal including adequacy components (with highest possible score for each shown) of total fruit (5), whole fruit (5), total vegetable (5), greens and beans (5), whole grains (10), dairy (10), total protein foods (5), seafood and plant proteins (5) and fatty acids (10) and moderation components of refined grains (10), sodium (10), added sugars (10) and saturated fats (10). Adequacy components are positively scored, where a higher intake results in an increased score, while moderation components are reversed scored, where a lower intake results in an increased score. Therefore, the total HEI score ranges from 0 to 100 with higher scores reflecting a higher diet quality and a score of 80 reflecting a high-quality diet among preschool aged children [93]. In the context of this study, we assessed diet quality to reflect dietary intake during childcare, and not with the assumption that this was indicative of overall dietary intake. Previous studies have used DOCC observations and HEI to assess children’s dietary intake over less than a full day to depict dietary intake in the childcare setting [54, 94, 95] and in school lunch [96], and to measure children’s dietary change in childcare [63].

Children’s PA was measured using accelerometer data (Actilife software, Actigraph) [92], using appropriate cut points and energy expenditure formulas for 2–5 year old children [97, 98]. Minimum wear criteria (i.e., ≥1 day of wear, ≥3 h of wear during the FCCH day) were established [96]. Five-second epochs were used to better detect short bursts of PA, and Freedson et al. cut-points cut-points for this age group were used to categorize activity into sedentary, light, moderate, vigorous, and moderate-to-vigorous [MVPA] activity based on METs [97, 98]. Time filters were based on the times that research staff recorded affixing and removing the accelerometers each day, as well as the times when the children began and ended their nap times. Actigraph wear time averaged per day was 5.6 h (1 h to 9.5 h), and the median wear time was 6.3 h. These wear times were adequate to capture PA during the childcare time period, which was the only interest of this study.

Day-level data for each child were averaged over the 2 days, then standardized into minutes per hour to account for variation in the length of the FCCH day and children’s wear time and then averaged at the FCCH level. Data were scored to create variables associated with time (and percent of observed time) each child spends in sedentary, light, moderate, and vigorous activity across the two-day observation period, not including naptime. Primary PA outcomes were: time (in minutes) and percent time in sedentary and MVPA.

### Statistical analysis

The proposed sample size of 60 FCCHs per condition was based on power of 0.8 or greater and alpha of .05, which allowed the study to detect effect sizes of at least 0.25 serving of fruits and vegetables per day and 3 oz. of 100% fruit juice or sugary beverage, as well as an increase of 2.1% time in MVPA [64]. To account for attrition, we planned to recruit 66 homes in each experimental group (total *n* = 132).

Descriptive statistics of frequencies and means and standard deviations are presented for FCCP characteristics and the mean scores for HEI-2015 and PA at baseline and month 8. Generalized Estimating Equations (GEE) with robust standard errors were used to model HEI scores and PA over time. A working independence correlation structure was used to correct for dependency of observations within FCCP. We examined intervention effects both with and without adjustment for FCCP ethnicity. Statistical significance was set at 2-tailed α = 0.05. All analyses were conducted in SPSS version 24 [99].

## Results

**Fig. 2:**
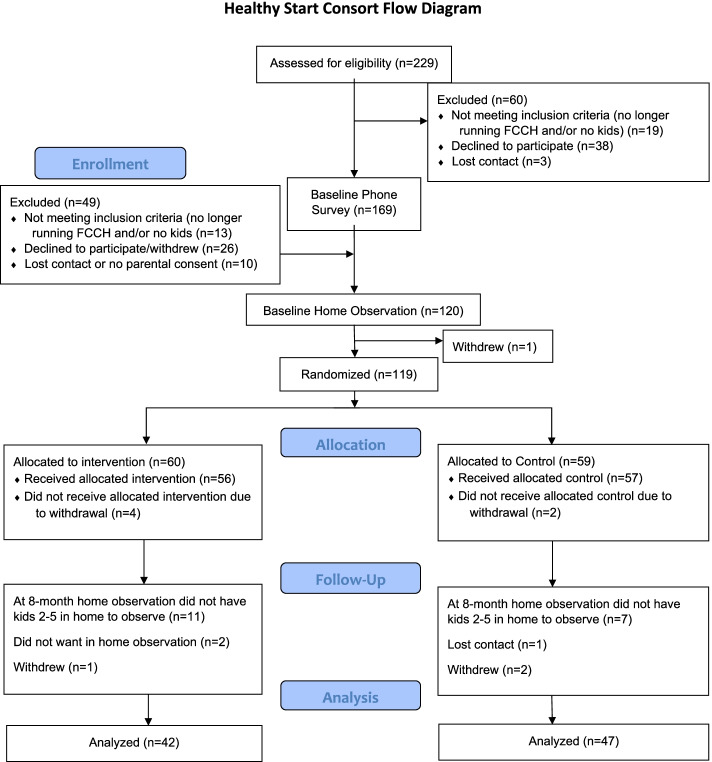
Healthy Start Consort Diagram

Enrollment and baseline data collection began in October 2015 and follow-up data collection ended in June 2019. Recruitment resulted in 168 FCCP who completed the baseline survey, 126 completed the in-person survey/baseline visit and consented to enroll, and 120 completed the two-day baseline observations. Of those, one FCCP withdrew after baseline measurement but before randomization. The Consort diagram is shown in Fig. 2. A total of 423 parents of eligible children consented for their children to be observed and/or measured; 377 of those children had at least one measurement; 370 children had their meals observed; 349 had accelerometer measurement, and 327 had anthropometric measurements. In relation to sample size goals [64], we randomized 90% of 132 targeted FCCP (*n* = 119) and 95% of 396 targeted children (*n* = 377).

Demographics of participating FCCP are shown in Table 1. There were no significant differences in measured demographic characteristics by experimental condition. FCCP averaged 49 years of age. Most were married (77%), self-identified as Latinx (67%) and as white (66%), were born outside the U.S. (70%), and lived in the U.S. for an average of 23 years at time of data collection. Few FCCP (18%) had attained a college education with 43% having less than a high school education. FCCP had an average of 12.8 years of experience in early child education. They cared for an average of 7.7 children, and most (82%) participated in the Child and Adult Care Food Program (CACFP).

**Table 1:**
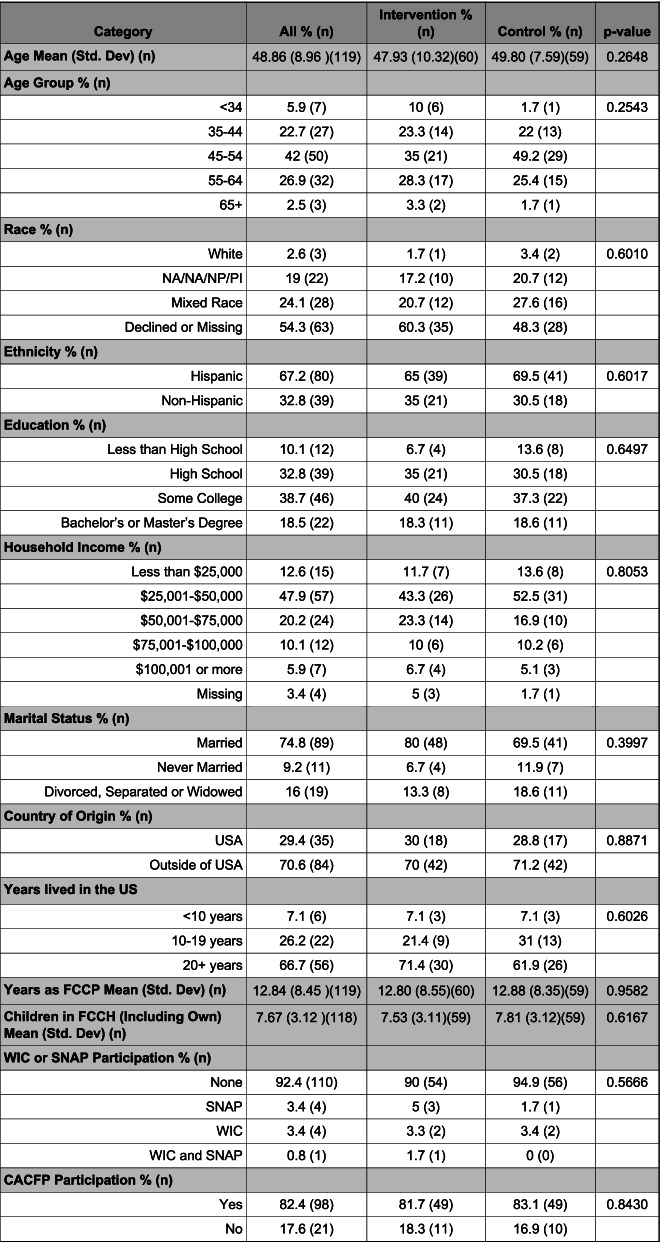
Demographics of FCCP participating in Healthy Start/Comenzos Sanos by Experimental Condition

Demographics of participating children are shown in Table 2. About half (51%) of the children were female and the mean age was 42 months (3.5 years). More than half (58%) of the children were Latinx, and 45% were white. Most (74%) of the children spent 8 or more hours at the FCCH on the days they attended and most ate both breakfast and lunch at the FCCH (85 and 97% respectively). Over a third (34%) of the children were classified in the overweight or obese categories [75], which is higher than national averages [100].

**Table 2:**
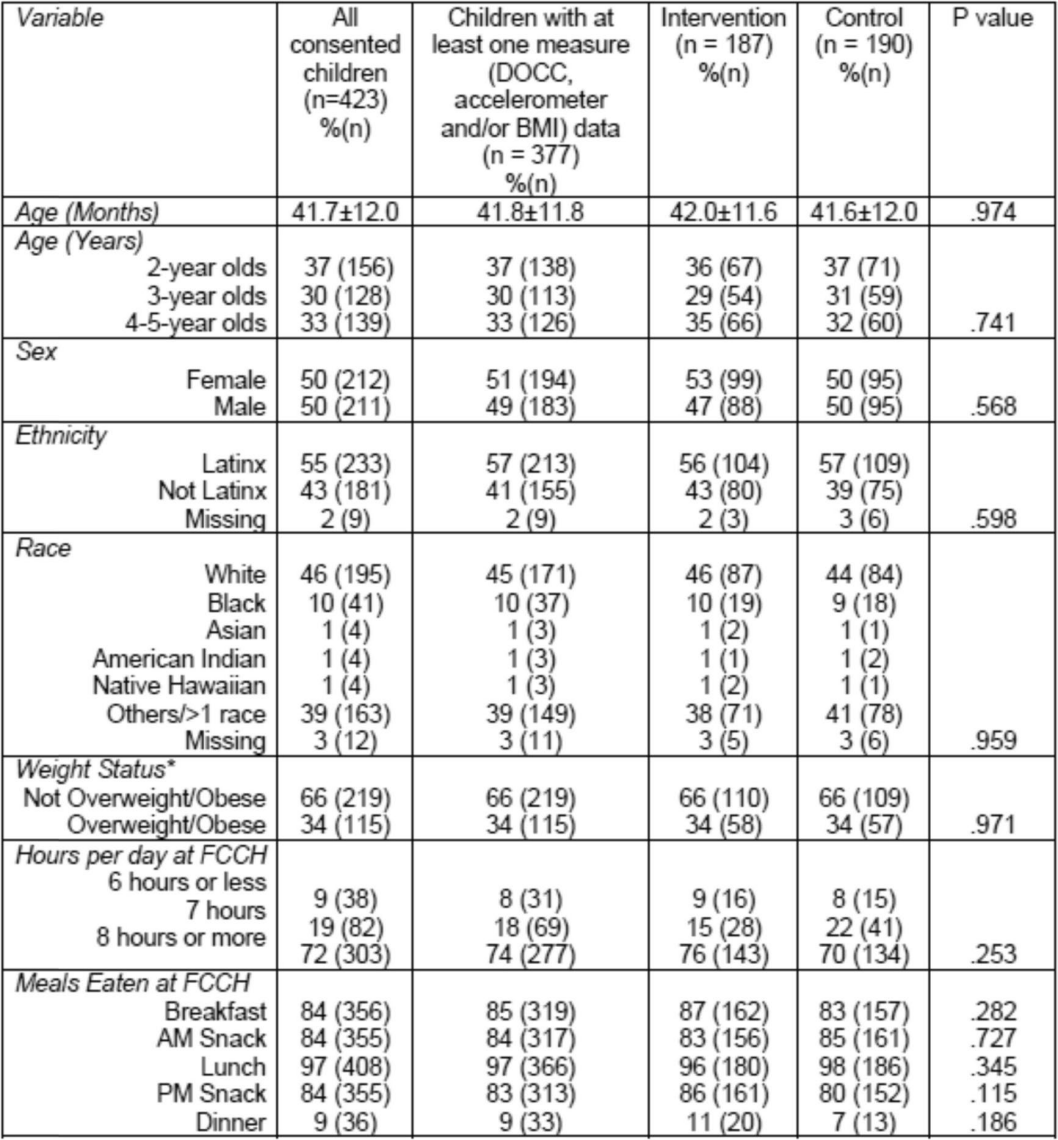
Demographic characteristics of children whose parents consented for participation in the Healthy Start/Comienzos Sanos Randomized Controlled Trial and children participating in data collection by Experimental Condition

### Process evaluation results

All FCCP completed the in-home coaching visit with 82% completing all 7 support coach telephone calls; 94% reported that the coach helped them to make changes and 90% reported that the coach was very helpful. Over 80% of FCCP (83%) reported reading all 8 tailored newsletters, with 87% reporting that they were very helpful; 58% reported watching all 8 tailored videos with 89% watching at least half of them; 82% said that the videos were very helpful. Regarding the active toys, 86% of FCCP said that the toys were very helpful. FCCP reported that they used the toys a median of 7 times per week, and the activity cards a median of once per week. Most (84%) of providers rated the activity cards as very or somewhat helpful. The majority (60%) of FCCP watched all six of the toy video segments with 69% watching at least half. When FCCP were asked which toys have been favorites with the children, the most frequently reported was the hula hoops, mentioned by 69% of FCCP. However, 58% of FCCP reporting that they attended no group meetings and only 12% of FCCP attended at least half the group meetings. Of those who did attend the meetings, 91% said that they were very or somewhat helpful.

### Primary outcome results: child diet quality

Table 3 shows changes in children’s diet quality as measured by the HEI-2015 total and component scores from baseline to 8 months by experimental condition averaged at the level of the FCCH. Total HEI-2015 scores increased by 3.8 points for the intervention group while scores decreased by 3.3 in the comparison group for an effective difference of 7.2 points, *p* < 0.001. HEI-2015 component scores for total vegetables increased by 0.5 points for the intervention group, while scores decreased by 0.4 points in the comparison group for an effective difference of 0.9 points, *p* = 0.025. Further, HEI-2015 component scores for added sugar increased by 1.0 points for the intervention group while scores remained the same for the comparison group, for an effective difference of 1.0 points, *p* = .025. HEI-2015 scores for refined grains increased by 1.3 points for the intervention group while scores decreased for the comparison group by 0.2 points for a difference of 1.5 points, *p* = 0.054. Greens and beans, whole grains, and total protein also showed trends in a positive direction favoring the intervention group. Other components scores showed no differences.

**Table 3:**
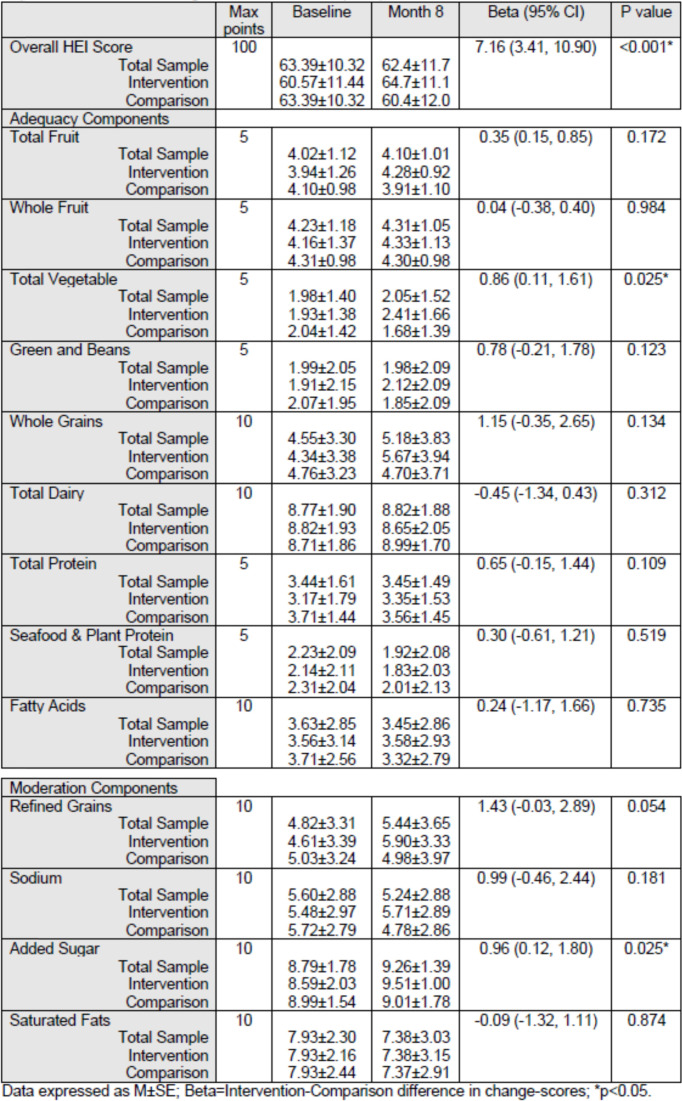
Change in Healthy Eating Index-2015 scores from Baseline to 8 Months by Experimental condition; Longitudinal associations (GEE)

Table 4 shows changes in children’s PA in the FCCH from baseline to 8 months by experimental condition as measured by accelerometer. From baseline to 8 months, children in the Intervention FCCH decreased their sedentary time by 1.8% while children in the Comparison FCCH increased their sedentary time by 3.9% for a difference of 5.7%, *p* = .021. From baseline to 8 months, children in the Intervention FCCH increased their time spent in MVPA by 1.5% while children in the Comparison FCCH decreased by 0.5% for a difference of 2.0%, *p* = 0.068, which was not statistically significant, but demonstrates a trend in a positive direction. Opportunities to observe outdoor play did not differ between experimental groups (data now shown). Over 98% of FCCH in both groups had outdoor observations. Thus, group differences were not due to issues such as varying weather.

**Table 4:**
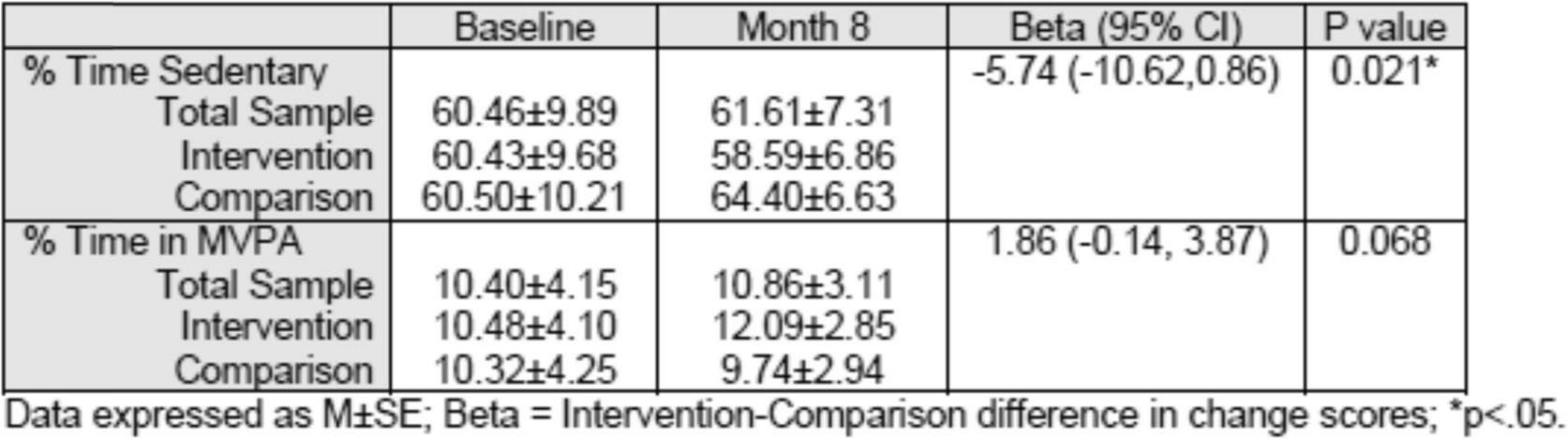
Changes in Percent of children’s time spent in Sedentary Behavior and MVPA from Baseline to 8 Months by Experimental condition; Longitudinal associations (GEE)

## Discussion

The multi-component Healthy Start/Comienzos Sanos intervention, which included lay support coaching, tailored feedback, print and video materials and group meetings, was efficacious in improving overall diet quality of preschool children in FCCH, including increasing vegetable and decreasing children’s added sugar intake. In addition, the intervention decreased sedentary time of children in FCCH. Thus, the Healthy Start study adds to the very limited pool of obesity prevention intervention studies conducted in FCCH, and is among the first to use a cluster randomized trial and to examine intervention effects on child outcomes.

Very few intervention studies aiming to improve the food and PA environment within FCCH have been conducted [50, 63]. In a quasi-experimental study, Trost et al. evaluated the Healthy Kansas Kids train-the trainer intervention with FCCH in 15 Kansas counties and demonstrated improvements in self-reported FCCP diet and PA practices, but this study did not measure diet or PA at the child level [62]. The Romp and Chomp quasi-experimental study in Victoria, Australia found that children in FCCH that received a multicomponent community-wide intervention spent less time watching television and using computer/electronic games, less time in organized active play and less time in free inside play compared with the comparison sample [59]. The Romp and Chomp intervention FCCH also had better nutrition environments than comparison FCCH, but this study used an environmental audit and did not measure child level nutrition or PA behavioral outcomes.

To our knowledge, only one other cluster randomized trial in the FCCH setting has reported child level outcomes. Keys to Healthy Family Child Care Homes [63, 64, 101] published its outcome findings in 2020 [63]. The aims of this study, conducted in North Carolina, were to improve children’s diet quality (2 days of observed intakes using DOCC) and PA (3 days of accelerometry) at the FCCH. This study enrolled 166 FCCH providers and 496 children aged 1.5–4 years. The 9-month intervention was delivered by paraprofessionals through three workshops, three home visits, and nine phone calls and addressed the FCCH diet and activity environment, provider health, and business practices. The attention control (comparison) arm received a business-focused intervention only. While both the Keys and Healthy Start studies were conducted in FCCH, Keys recruited FCCP that were predominantly African-American (74%) while our Healthy Start study had mainly Latinx (67%) FCCP. Similar to Healthy Start, the Keys intervention children significantly increased total HEI-2010 scores relative to comparison children (+ 5.39 points, *p* < 0.001), which is slightly smaller than the HEI-2015 changes observed in our Healthy Start study (+ 7.2 points, p < 0.001). The Keys study also observed significant improvements in several HEI-2010 component scores (whole grains, seafood/plant protein, refined grains, and sodium, for all *p* ≤ .031), but noted a significant decrease in total vegetable score (− 0.49 points, *p* = .003). In contrast, our Healthy Start study noted significant improvements in total vegetable scores (+ 2.5) and added sugars (+ 2.4). In the Keys study, no significant differences were noted between study arms for changes in children’s MVPA, active play, or sedentary time, while the Healthy Start study did find significant differences between study arms in sedentary behavior favoring the intervention group (− 5.7%). Using similar study designs and measures, the Healthy Start study was able to achieve slightly better outcomes.

Reasons for the different findings between Keys and Healthy Start might be speculated. In addition to the population differences mentioned above, unlike Keys, Healthy Start included active play toys together with video segments and activity cards on how to use the toys, which may have helped the providers to decrease the sedentary time of the children. Characteristics of FCCH that are different from childcare centers can make PA difficult including single caregiver environments (or fewer staff members), caring for children of wider age ranges, competing demands on provider time (e.g. cleaning, paperwork, cooking), less space for PA and lower levels of staff training [40, 50, 102]. FCCP may also have lower personal self-efficacy for PA as well as inactive lifestyles and health issues, which may impact their ability to participate with children in PA or be positive role models [45, 50, 103]. Future interventions should strengthen the PA components of the intervention beyond both the Keys and Healthy Start models by, for example, providing FCCP with baseline PA levels of children and which activities result in highest PA levels, technical assistance for reorganizing FCCH indoor environments to allow for more gross motor activities [63, 104], providing specific training in provider-led PA, scheduling daily indoor dance parties, offering active screen time resources that help engage children of different ages in MVPA [63, 105], and/or addressing safe PA opportunities in the FCCH neighborhood [106, 107]. These recommendations concur with Jones et al., who in reviewing childcare PA interventions, concluded that future interventions need to consider creative ways of delivering childcare-based PA interventions [108].

The 5.4 and 7.2 HEI point improvements in children’s diet quality for the Keys and Healthy Start studies, respectively indicate the potential for dietary interventions in FCCH. The results from both studies are clinically meaningful as an increase in 5 HEI units predicts a 4–6% decrease in overall mortality [109–111] and a 15% decrease in the prevalence of obesity.^109110111^ Previous research among toddlers outside the childcare setting has shown differences in HEI ranging from 0.09 for every month delay in juice and or sugar sweetened beverage introduction [112–114]. In the family-based LAUNCH obesity program, Robson et al. achieved a 7.0 increase in preschooler HEI scores compared with standard care (Robson 2019). In addition, early childcare nutrition policy changes in South Carolina (compared with North Carolina, where no policies were changed) resulted in HEI component changes (whole fruits, total fruits, and lean proteins, but decreased scores for dairy), but not overall HEI change [112].

Both the Keys and Healthy Start studies provided technical assistance based on NAP SACC guidelines, but Healthy Start delivered an 8-month intervention using peer support coaches to deliver MI via one visit and 7 telephone calls and provided tailored newsletters and videos, in contrast to the 9-month Keys intervention, which was delivered by paraprofessionals through three workshops, three home visits, and nine phone calls. A recent umbrella review of nutrition interventions in childcare settings found that effective interventions were led primarily by external experts, which may not be cost-effective/sustainable [64]. The support coaches in the Healthy Start study were bilingual trained community health workers selected to have prior childcare experience. They were particularly skilled at developing a rapport with FCCP, well liked and considered very helpful, and are less expensive than employing professionals and paraprofessionals. Thus, community health workers have promise for delivering future interventions in childcare settings including FCCH.

The majority of FCCP in Healthy Start were participants in CACFP. Federal CACFP food requirements were strengthened in 2017 during the Healthy Start study; however, improvements in children’s diet quality during the study were not due to these policy changes as there were no differences in CACFP participation by experimental group. However, interventions to improve child diet quality might gain more traction when FCCH participate in the CACFP. Several studies in FCCH have shown that providers in CACFP are more likely to have a written nutrition policy [115]; serve more nutritious foods [115–117]; and engage in more best nutrition practices compared to non-CACFP providers [117–120]. Interventions within FCCH should strongly encourage eligible providers to enroll in the CACFP and other food safety net programs if they are eligible [121, 122]. A recent study found that the CACFP meal reimbursement rate was related to FCCP’ perceptions of the adequacy of the reimbursement and the difficulty of purchasing qualified foods [123]; thus, future interventions might be more successful if they include components that provide training for FCCP on food budgeting and lower costs meals and snacks [124]. Furthermore, federal policy makers should consider increasing CACFP reimbursement rates and further strengthening CACFP nutrition guidelines and/or state licensure guidelines to bring them more in line with the NAP SACC best practices.

A recent umbrella review of nutrition interventions in childcare settings highlighted the need for increasing collaborative parental involvement and engagement in childcare interventions as more parental engagement has been related to more positive child dietary changes [125]. Interventions delivered in childcare settings have rarely involved parents in a substantial or effective way to influence both important child environments (childcare and home) [126, 127]. Given the shared responsibility between parents and childcare providers for children’s health, shared communication regarding promotion of healthy eating and activity may be beneficial in supporting consistent health-related messages for young children [128]. However, communication between parents and FCCP related to preschool-aged children’s health-related behaviors remains limited [67]. Healthy Start baseline data found that over half of FCCP reported that they provided families with information about appropriate screen time behaviors for children [129], but less than 40% said that they provided families with information on PA or nutrition [65, 129]. Research suggests that highly trained FCCP are more likely to disseminate obesity prevention information to both children and parents [130]. Thus, there may be promise in providing training and technical assistance to FCCP so they can more effectively engage parents [50, 102] to promote a healthy nutrition and activity environment both in childcare and at home, which can facilitate children’s healthy eating and PA across the entire day to prevent obesity [69, 70, 131]. Future studies may also consider collecting diet, PA, screen-time data both in the childcare and home setting to more comprehensively capture usual behaviors across these settings. This would allow for future studies to measure whether childcare interventions that improve children’s diet and/or PA in childcare have crossover effects to the child’s diet and PA at home, and studies to explore innovative ways to engage parents in childcare interventions.

Healthy Start process data show that most of the intervention components (e.g. support coach visits/calls, written materials, videos, active toys) were delivered with high fidelity and were well received by FCCP. However, the group meetings were not well attended, even though FCCP did express a desire and need for social support with other FCCP in our formative work [69, 70]. Even though we tried to schedule these meetings at evening and weekend times and in convenient locations, FCCP found them difficult to attend, although those who did attend, found them to be helpful. Other studies have found that barriers to attending in person training include schedule conflicts, accessibility, and cost [132]. Future interventions should consider offering support groups using virtual methods like video conferencing and/or social media approaches. Research has shown that FCCP are open to online trainings [132].

These results also highlight several areas for future research. First, mediational pathways could be explored including examining changes in FCCP’s psychosocial determinants (e.g., attitudes, perceived barriers, self-efficacy) due to the intervention and how those relate to changes in their practices and in turn children’s behaviors. In addition, analyses could examine moderators of change, which might include length of time serving as FCCP, or aspects of the physical environment, such as FCCH size, that are difficult to change. Future studies should also include younger children (birth to 2 years) in addition to preschool children as many infants and toddlers are also cared for in FCCH, yet rarely included. Our observations in Healthy Start and other pilot work [133] demonstrate a need for interventions to improve FCCP’s feeding practices, time in active play and screen time for infants and toddlers.

## Limitations

In consideration of these findings, it is important to note several study limitations. The sample of participating FCCP are likely not completely generalizable to the overall population of FCCP in this geographic location primarily due to their willingness to participate in research. Additionally, all FCCP in this study were women and most were Latina, which excludes providers of other ethnic backgrounds and the few men who run such businesses. Another study limitation was the moderately high attrition, caused primarily by child enrollment turnover. However, FCCP and children who completed follow-up were similar to those who were lost to follow-up, suggesting that attrition did not overly bias the sample. In addition, there was no differential dropout between experimental groups, therefore we did not include imputation of missing values in the analyses. Though our recruitment approached our goals based on initial sample size calculations, stronger recruitment and retention would have strengthened our ability to scientifically observe meaningful differences between groups. In addition, we did not measure whether blinding of observers was successfully achieved. However, the field staff were not told the experimental group of FCCH they observed. Toys that were provided did not have any project identifying information on them, so should not have unblinded experimental group status. In addition, the observers were trained not to have conversations with providers except to clarify information about recipes or ingredients.

The intervention itself was multi-component and allowed providers to choose from a wide variety of topics, which offered choice, but also meant that we delivered somewhat different intervention content among participating FCCP, limiting our understanding of intervention mechanisms. Future studies could conduct dismantling implementation research to determine which intervention components are most predictive of behavior change and/or design more of a core intervention with less tailoring or focus tailoring on most needed topics.

Furthermore, though the observational measure of diet and the accelerometer measure of PA are state-of-the-art for childcare research, only two assessment days of diet and PA may not capture the variability of children’s dietary intake and PA. Because the days of observation were announced, it is possible that FCCP changed their behavior on the occasions of having observers in their home, although this would likely have affected both experimental groups. Also, the study evaluation only included follow-up at 8 months, which was immediately after the end of the intervention. This timeline precluded assessment of sustainability of the children’s behavior changes. Future studies could include a second longer term follow-up.

## Conclusions

In conclusion, the multi-component Healthy Start intervention in FCCH improved preschool-aged children’s diet quality in childcare including increased vegetable intake and decreased added sugar intake. The intervention also decreased the time that children spent sedentary in childcare, and showed a trend toward increasing MVPA. The Healthy Start study reinforced the previous literature, which indicates that FCCH are in need of dietary and PA interventions and that such interventions are well accepted by childcare providers and can be successful in improving children’s behaviors in childcare, especially diet quality [59, 62, 63]. As US children attending FCCH spend on average 31 h per week in this setting [42], and most children in Healthy Start spent 8 h or more on the days they were in childcare, FCCH are important settings for supporting children’s diet and PA. Given the ongoing need for primary prevention of childhood obesity and the important role that FCCH environments play in impacting the health behaviors among low-income families, more research is needed to better address obesity prevention in this setting with the eventual goal to disseminate effective FCCH-based interventions into childcare systems such as CACFP and FCCH networks.

## Supplementary Information

**Additional file 1.**

## Data Availability

The datasets used and/or analyzed during the current study are available from the author Risica on reasonable request.

## Abbreviations

PA: Physical Activity
FCCH: Family Childcare Home
FCCP: Family Childcare Provider
CACFP: Child and Adult Care Food Program
HEI: Healthy Eating Index
MV: Moderate to Vigorous
