# Supplementary material for: A multi-component tailored intervention in family childcare homes improves diet quality and sedentary behavior of preschool children compared to an attention control: results from the Healthy Start-Comienzos Sanos cluster randomized trial
Source: Int J Behav Nutr Phys Act. 2022 Apr 15;19:45. doi: 10.1186/s12966-022-01272-6 (PMC9013065; doi:10.1186/s12966-022-01272-6)

Appendix 1. Sample pages from the tailored feedback form

About your child care home…

[Provider's first name], when you answered our survey, you gave us information about how things run in your home. We also gathered information during the days we spent with you in your home.

With this report, you can see where you are already doing well: ✓

And where you could make a change: no check mark.

HEALTHY DRINKS

BEST PRACTICE:	IN YOUR HOME:	✓
**WATER**<br><br>Make drinking water available for children at all times.	We observed you *[had OR did not have]* drinking water available at all times.	
Prompt children to drink water during each indoor and outdoor play time.	We observed you [always / did not always] encourage children to drink water during active playtimes.	
**JUICE**<br><br>Limit 100% fruit juice to no more than two, 4-6 ounce servings per week.	You said you offer 100% juice to children ___ times a [day/week/month] and the serving size you usually offer is ____ ounces.<br><br>We observed you served more than the recommended amount of juice.	
Only serve 100% fruit juice that has no sugar added.	We observed you [did / did not] serve juice that had added sugar.	

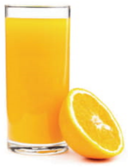

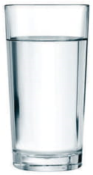

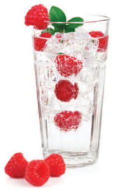

HEALTHY FOODS

BEST PRACTICE:	IN YOUR HOME:	✓
**FRUIT**<br>Offer children fruit two or more times a day.	We observed you served fruit ___ times a day.	
Never serve fruit in syrup or with added sugar.	We observed you [did / did not] serve fruit in syrup or with added sugar.	
**WHOLE GRAINS**<br>Offer children high fiber, whole grain foods two or more times a day.	We observed you served whole grains ____ times a day.	
**HEALTHY SNACKS**<br>Limit offering children sugary, salty, or fatty foods to less than 1 time per week or never.	You said you serve **sugary** foods ____ times per [day/ week/month].<br><br>You said you serve **salty or fatty** snack foods ____ times per [day/week/month].	
	We observed you serve **sugary** foods ___ times during our visit.<br><br>We observed you serve **salty or fatty** snack foods ____ times during our visit.	
**HIGH FAT MEATS**<br>Limit serving high-fat meats to less than 1 time per week or never.	You said you serve high-fat meats (such as sausage, bacon, hot dogs, bologna) ___ times per [day/week/ month].<br><br>We observed you serve high-fat meats ___ times during our visit.	

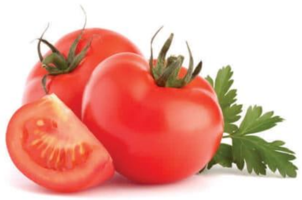
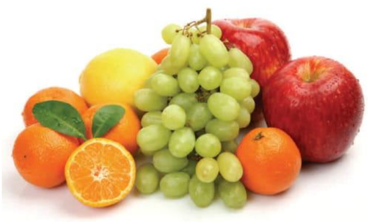
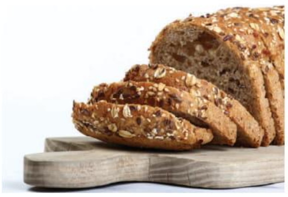

PHYSICAL ACTIVITY

BEST PRACTICE:	IN YOUR HOME:	✓
**PHYSICAL ACTIVITY**<br><br>Provide children with 90 minutes or more of indoor or outdoor physical activity each day.	We observed children had ___ minutes of indoor or outdoor physical activity a day.	
**OUTDOOR PLAY**<br><br>Provide children with 60 minutes or more of outdoor play each day.	We observed children play outside for a total of _____ minutes each day.	
**ADULT-LED PHYSICAL ACTIVITY**<br><br>Provide children with 45 minutes or more of adult-led physical activity each day.	We observed children had ____ minutes of adult-led physical activity each day.	
**PHYSICAL ACTIVITY EDUCATION**<br><br>Lead a planned physical activity class 1 or more times per **week**.	You said you lead planned physical activity lessons [rarely or never, 1 time per month, 2-3 times per month, twice a week or more].	
Talk with children informally about the importance of physical activity.	We observed [you did/ did not] talk informally with children about the importance of physical activity.	

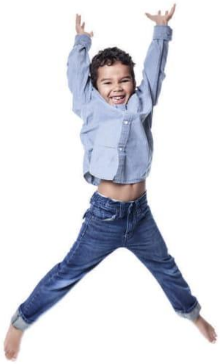
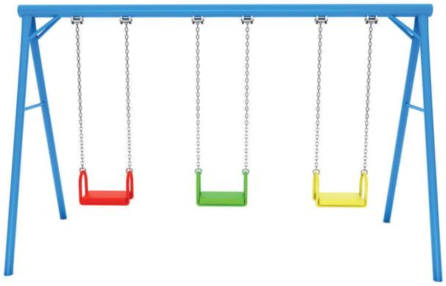
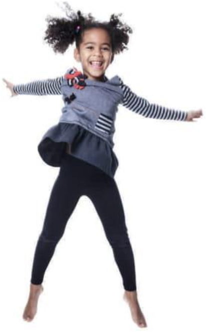

Supplement: Supplementary file 1 — Additional file 1. [file 12966_2022_1272_MOESM1_ESM.pdf]
